# Lidocaine-Loaded Thermoresponsive Gel for Accelerated Wound Healing in Dry Socket and Oral Wounds

**DOI:** 10.3390/gels10110739

**Published:** 2024-11-14

**Authors:** Nuttawut Supachawaroj, Kunchorn Kerdmanee, Sucharat Limsitthichaikoon

**Affiliations:** 1 Department of Oral Surgery, College of Dental Medicine, Rangsit University, Pathum Thani 12000, Thailand; nuttawut.s@rsu.ac.th; 2 Department of Periodontics, College of Dental Medicine, Rangsit University, Pathum Thani 12000, Thailand; kunchorn.k@rsu.ac.th; 3 Department of Pharmaceutical Technology, College of Pharmacy, Rangsit University, Pathum Thani 12000, Thailand

**Keywords:** dry socket wound healing, in vitro wound scratch assay, thermoresponsive gels, polyelectrolyte complex, palatal wounds

## Abstract

Dry socket, also known as alveolar osteitis, presents significant challenges in oral surgery because of severe pain and delayed wound healing. This study aims to address these challenges by developing and evaluating a lidocaine-loaded polyelectrolyte complex thermoresponsive gel (LG) designed to enhance wound healing and provide effective pain management in oral wounds. The thermoresponsive gel transitions from a liquid to a gel at body temperature, ensuring sustained contact with the wound site and prolonged release of lidocaine. The in vitro assessments, including cytotoxicity and wound scratch assays, demonstrated the biocompatibility and therapeutic potential of the LG formulation. Following this, palatal wounds were induced in rats, with healing monitored over a 14-days period. Histological analyses were conducted to assess tissue regeneration and inflammation. The results indicated that the LG formulation significantly improved wound closure rates, reduced inflammation, and accelerated epithelialization compared with control groups, primarily because of the high content of hyaluronic acid (HA). The synergistic effects of HA combined with the thermoresponsive properties of the gel facilitated faster healing. These findings suggest that LG is a promising therapeutic option for enhancing oral wound healing and effectively managing pain, particularly in conditions such as dry socket.

## 1. Introduction

Dry socket, or alveolar osteitis, is a well-known and highly painful complication that can occur following tooth extractions, particularly molar extractions [1,2]. This condition arises when the blood clot that forms naturally at the extraction site—crucial for protecting the underlying bone and nerves—is dislodged or dissolves prematurely [3,4]. The exposure of bone and nerve endings leads to severe pain, often described as throbbing or radiating, which can persist for several days [1,2,3]. Additionally, the absence of a protective blood clot delays the wound-healing process, leaving the extraction site vulnerable to infection and further complications [2,5,6,7]. The management of dry socket primarily focuses on two key objectives: alleviating pain and promoting effective wound healing [8,9]. While topical analgesics can provide targeted pain relief at the wound site, pain may still linger for days, significantly impacting the patient’s quality of life [8,9,10]. The typical healing process takes approximately 5 to 7 days, during which effective pain management is essential [5,11,12,13]. However, existing treatments often fall short of delivering both immediate relief and accelerated healing, revealing a critical gap in current oral wound management strategies. This research aims to bridge that gap by developing a treatment that not only offers sustained pain relief but also enhances wound healing in dry socket conditions.

Topical lidocaine is widely recognized for its rapid and localized pain relief in oral wounds, minimizing the systemic side effects commonly associated with oral analgesics [14,15,16]; however, maintaining consistent contact between lidocaine and the wound presents challenges in the moist and dynamic environment of the oral cavity, where saliva flow and mechanical forces from eating and speaking can disrupt treatment [16,17,18,19]. Lidocaine-loaded thermoresponsive gels offer a promising solution to this issue. These gels remain liquid at lower temperatures and transform into a gel at body temperature, allowing them to adhere effectively to the wound site [16,17,19,20,21]. This phase transition ensures sustained contact with the wound, prolonging the release of lidocaine and providing extended pain relief. Moreover, the gel acts as a protective barrier, supporting tissue regeneration.

By combining lidocaine with thermoresponsive gels, we address the immediate need for pain control while also focusing on the longer-term goal of accelerated healing [10,22,23]. Additionally, these gels not only facilitate controlled and prolonged drug release but also help minimize irritation and inflammation, ensuring better wound coverage and a more favorable healing environment [24,25,26,27,28]. When integrated with agents such as hyaluronic acid, the gels further enhance tissue regeneration and reduce inflammation, thereby improving the overall healing process [10,14,28]. The use of a palatal wound model in rats is a practical and reliable method for evaluating these treatments. This model closely mimics the human oral cavity in terms of blood supply and exposure to mechanical stress, making it ideal for studying the effects of wound therapies [29,30,31]. It allows for controlled, reproducible studies, providing detailed insights through histological analysis on the effectiveness of gels in wound healing and inflammation management.

In this study, we evaluate the effectiveness of a lidocaine-loaded polyelectrolyte complex thermoresponsive gel (LG) in promoting palatal wound healing using a rat model. We focus on the gel’s ability to accelerate wound healing in both in vitro and in vivo settings. In vitro tests, including cytotoxicity and cell migration assays, were conducted to assess the gel’s biocompatibility and efficacy. In vivo wound healing in the rat palatal model, coupled with histological analysis, provides deeper insights into the gel’s performance in a biological context. The findings of this research could have the potential to inform improved clinical practices for managing dry socket wounds, ultimately enhancing patient recovery and care.

## 2. Results and Discussion

### 2.1. Liodocaine Loaded Thermoresponsive Gels Characteristic

Table 1 presents the composition of the blank control gel (BG) and the LG formulations. All thermoresponsive hydrogels appeared as white, turbid, homogeneous viscous solutions at room temperature, exhibiting a pH range of 6.8–7.0. Although varying concentrations of hyaluronic acid (HA) did not alter the appearance of the formulations, they significantly increased viscosity.

The gelation temperature of the LG formulations ranged from 26–28 °C, with a gelation time of approximately 2 min. This indicates that the gels are capable of forming at oral cavity temperatures, which typically range from 35 °C to 37 °C [32,33,34]. The average temperature of the human oral cavity is approximately 35.5 °C to 37.5 °C, slightly lower than core body temperature because of factors such as airflow, saliva evaporation, and the consumption of food and liquids. Consequently, our formulations remain in a liquid state during storage and application but rapidly transition to a gel upon contact with the oral mucosa [32,33,34]. This unique property ensures proper adherence to the wound site and allows for sustained drug release, thereby enhancing the overall effectiveness of the treatment.

All formulations contained lidocaine levels ranging from 100% to 106% of the labeled amount (%LA), which is consistent with the acceptable range established by USP guidelines.

Prior to their application for in vitro cell scratch and animal studies, all tested formulations—including the LG, BG, lidocaine gel (LH), triamcinolone gel (TA), and hyaluronic acid gel (HA)—were evaluated for microbial contamination. The results indicated a total plate count of less than 10 CFU/mL and total yeast and mold counts of fewer than 10 CFU/g, with no detection of *Escherichia coli* or *Staphylococcus aureus*. These microbiological findings were well within acceptable limits, confirming the suitability of the formulations for subsequent animal and clinical investigations.

### 2.2. In Vitro Wound Healing

The formulations were evaluated on human gingival fibroblast (HGF) to assess their cytotoxicity and their ability to promote cell migration [35]. The cytotoxicity assay was conducted to determine the safety of the formulations for cellular applications, while the in vitro wound scratch assay evaluated the effectiveness of these formulations in stimulating fibroblast migration—a crucial process in wound healing [36,37]. Together, these tests provide valuable insights into both the biocompatibility and wound-healing potential of the formulations.

#### 2.2.1. Cytotoxicity Assessment

This study aimed to investigate the effects of each formulation on cell survival, thereby providing insights into the cytocompatibility of the gels. The cell viability of all formulations, including the blank gel (BG), was assessed using HGF. Cells treated with Dulbecco’s Modified Eagle Medium (DMEM) served as the negative control, representing normal cell conditions. In contrast, cells exposed to hydrogen peroxide (H_2_O_2_) were utilized as the positive control to induce oxidative stress and assess cytotoxicity. This comparative approach enabled us to evaluate the safety and biocompatibility of the formulations by observing their impact on cell viability in both healthy and stressed cellular environments.

**Figure 1:**
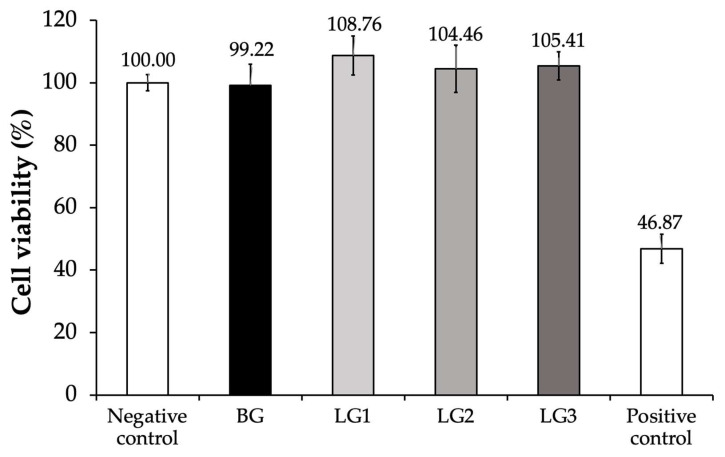
Effect of lidocaine-loaded thermoresponsive gel formulations (LG1-LG3) and blank control gel (BG) on cell viability (%) of Human Gingival Fibroblasts (HGF). Cells treated with DMEM served as the negative control, representing normal cell conditions, while cells exposed to hydrogen peroxide (H_2_O_2_) acted as the positive control. Error bars indicate standard deviations (*n* = 8).

The results (as shown in Figure 1) indicated that the blank gel (BG) did not compromise cell integrity and remained non-toxic to human gingival fibroblasts (HGF), similar to the lidocaine-loaded formulations (LG1, LG2, and LG3). In contrast, cells treated with hydrogen peroxide (H_2_O_2_), used as a positive control, exhibited significantly reduced viability, falling below 50% after 24 h of treatment. These findings suggest that the concentrations of lidocaine in our formulations (LG1, LG2, and LG3), along with the other ingredients in the gel, do not adversely affect HGF viability.

#### 2.2.2. In Vitro Wound Scratch

The percentage of viable cells across different formulations is illustrated in Figure 1. No significant differences were observed between the LG formulations and the BG when compared with the negative control. This indicates that all tested samples exhibited no detectable toxicity, suggesting that both the lidocaine-loaded gels and the blank gel are biocompatible and safe for use with human gingival fibroblasts. Consequently, the LG formulations were selected for further evaluation in the in vitro wound scratch assay.

**Figure 2:**
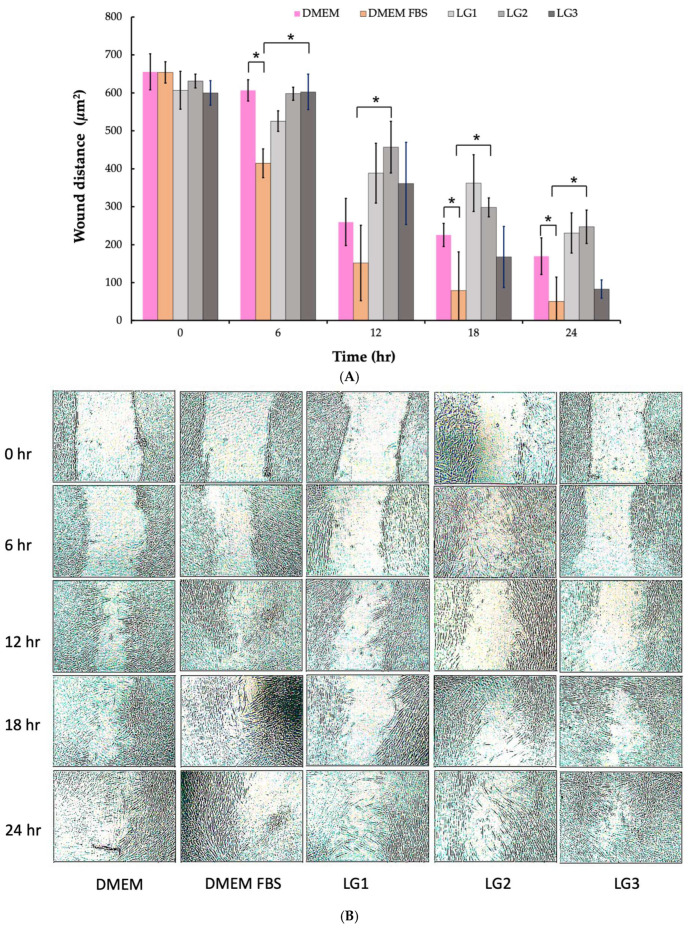
In vitro scratch assay of Human Gingival Fibroblasts (HGF). (**A**) Wound gap reduction (µm^2^) measured at 0, 6-, 12-, 18-, and 24-h post-scratch. (**B**) Images of cell migration in the scratch zones of HGF at the same time points. Cells treated with lidocaine-loaded thermoresponsive gel formulations (LG1-LG3) were compared with a DMEM-negative control, representing normal cell conditions, and a positive control group treated with DMEM-containing fetal bovine serum (FBS). Error bars indicate standard deviations (*n* = 8). Statistical significance (*) is denoted as *p* < 0.05 when comparing within groups.

The wound gap reduction (µm^2^) was monitored at 0, 6, 12, 18, and 24 h, as shown in Figure 2A, highlighting the progressive closure of the scratch zone. The rate of gap reduction provides insight into the cell migration capacity, with faster closure indicating enhanced wound healing potential [36,38,39].

At the beginning of the experiment, there was no significant difference in the wound gap area among the treatment groups; however, after 6 h, HGF cells treated with DMEM containing 10%FBS exhibited significantly faster migration, resulting in a reduced wound gap (*p* < 0.05) compared with the other groups. By 12 h, this trend persisted, with the DMEM + FBS group showing faster wound closure (*p* < 0.05), while no significant difference was observed between the negative control and LG3 group. At 18 and 24 h, both the DMEM + FBS and LG3 groups demonstrated significantly greater wound gap closure (*p* < 0.05) compared with the other treatments, with no notable difference between the two groups. This indicates that LG3 is as effective as the DMEM + FBS treatment in promoting cell migration and wound closure during the later stages of the assay.

The cell migration images presented in Figure 2B provide a visual representation of the healing process over time (0, 6, 12, 18, and 24 h), showcasing the progressive movement of HGF cells into the scratch zone. These images clearly demonstrate the gradual reduction in the wound gap, correlating with the ability of the tested formulations to promote cell migration and wound closure [38]. At each time point, the decreasing size of the gap reflects ongoing cellular activity, particularly the migration of HGF cells from the wound edges toward the center [40,41]. This visual evidence supports quantitative data, suggesting that the formulations effectively facilitate tissue repair by enhancing fibroblast motility, a critical factor in wound healing. By promoting wound closure in a time-dependent manner, these images further validate the role of the formulations in accelerating the cellular processes necessary for tissue regeneration [42]. This method of visual tracking not only underscores the therapeutic potential of the gels but also provides a clear and engaging demonstration of how the formulations interact with the biological environment to promote healing [40].

LG3, which has the highest concentration of hyaluronic acid (HA), significantly enhanced cell migration in the in vitro wound scratch assay, demonstrating its superior effectiveness in promoting wound closure [43,44,45]. This accelerated wound healing is directly attributable to the elevated HA levels in LG3, which support critical aspects of the healing process, including cell migration and proliferation, angiogenesis, inflammation regulation, hydration, and tissue regeneration. The high concentration of HA creates an optimal environment for rapid wound closure and improved tissue quality, resulting in a more pronounced reduction in wound size in the LG3-treated group compared with other formulations. These properties of HA not only facilitate the movement and growth of essential cells but also ensure sustained hydration and controlled inflammation, further enhancing the overall healing efficacy of LG3.

Importantly, LG3 did not induce any cytotoxic effects on HGF, confirming its safety for further application. The observed enhancement in cell migration can be attributed to the high concentration of HA, a biopolymer recognized for its role in tissue hydration, cellular proliferation, and wound healing [28,46,47]. By supporting the movement of fibroblasts into the wound area, LG3 accelerates the repair process, making it a strong candidate for therapeutic use. Based on these promising in vitro results, particularly its non-toxic nature and superior wound-healing properties, LG3 was selected for in vivo testing in a rat model.

### 2.3. Clinical Healing Analysis

This study aims to evaluate the therapeutic potential of the lidocaine-loaded gel (LG) formulation in promoting palatal wound healing using a rat model that closely mimics clinical conditions. Our research focuses on both the speed and quality of tissue repair, with the objective of demonstrating the ability of LG to enhance tissue regeneration, reduce inflammation, and accelerate the overall healing process. The rat model was chosen for its anatomical and physiological similarities to human oral tissues, making it an ideal platform for the preclinical evaluation of oral wound-healing therapies.

This study involved dividing the rats into five groups for a 14-day treatment period. Each group received different topical applications: blank control gel (BG), lidocaine-loaded thermoresponsive gel (LG), lidocaine gel (LH), triamcinolone gel (TA), and hyaluronic acid gel (HA). Wound sizes were measured on days 5, 7, and 14 and compared with the initial wound size to assess healing progress.

#### 2.3.1. Wound Size

**Figure 3:**
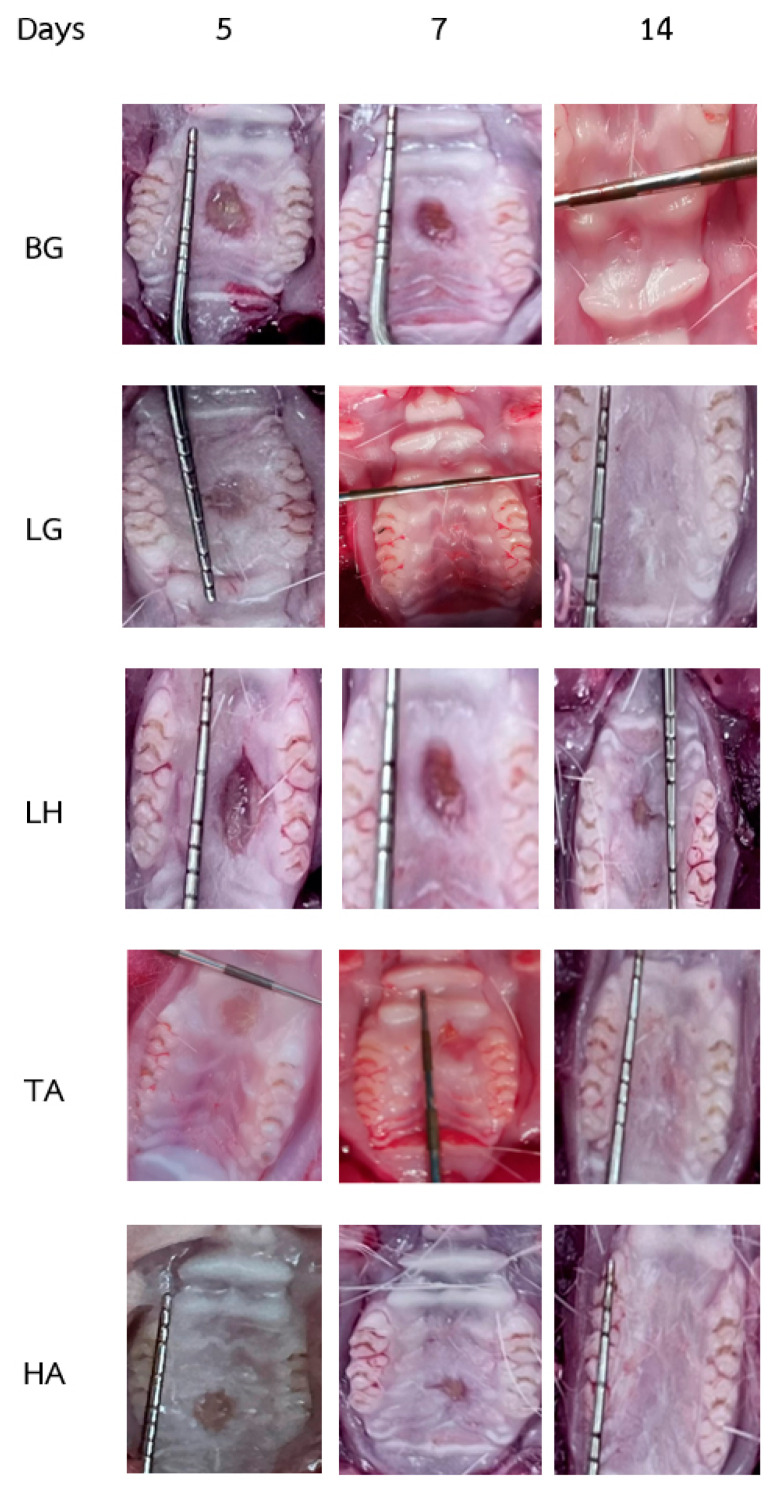
Wound size diameter (mm) of a wound after being treated with LG on days 5, 7, and 14. Displays a graph illustrating the reduction in wound size following the daily application of various gels over a 14-day period. The treatment groups included blank control gel (BG), lidocaine-loaded thermoresponsive gel (LG, lidocaine gel (LH), triamcinolone gel (TA), and hyaluronic acid gel (HA). Wound sizes were measured immediately after wound induction and subsequently on days 5, 7, and 14 to assess the healing progression across different treatments.

Wound size reduction was observed in all treatment groups, indicating that the natural wound-healing process was facilitated by the application of the various gels. As shown in Figure 3 and Table 2, the graph illustrates the progressive reduction in wound size across the different groups: BG, LG, LH, TA, and HA. The formulations were applied once daily for 14 days, with wound measurements recorded on days 1, 5, 7, and 14. The initial wound size, created using a 3 mm biopsy punch, was approximately 7.07 ± 0.1 mm^2^.

On day 5 of treatment, the LG group exhibited the most significant reduction in wound size, with the wound area measuring 4.27 ± 0.6 mm^2^. This reduction was significantly greater than the initial wound size of 7.07 ± 0.1 mm^2^ and compared with the other treatment groups (*p* < 0.05). The TA and HA groups also showed a notable decrease in wound size, measuring 5.98 ± 0.5 mm^2^ and 5.81 ± 0.4 mm^2^, respectively. In comparison, the BG and LH groups had wound sizes of 6.48 ± 0.4 mm^2^ and 6.42 ± 0.6 mm^2^, respectively, both larger than the wounds in the LG group.

Similar trends persisted on day 7, with the LG group maintaining the smallest wound size of 0.96 ± 0.3 mm^2^, significantly smaller than those in the other treatment groups (*p* < 0.05). The TA and HA groups continued to show substantial reductions in wound size, measuring 3.72 ± 0.3 mm^2^ and 3.49 ± 0.6 mm^2^, respectively. In contrast, the BG and LH groups measured 4.73 ± 0.7 mm^2^ and 4.83 ± 0.9 mm^2^, remaining larger than the wounds in the LG group.

By day 14, the wounds in both the LG and HA groups had completely healed, demonstrating the superior effectiveness of the LG formulation in promoting wound closure. In contrast, the BG, LH, and TA groups exhibited reduced wound sizes but had not yet achieved complete healing, dissimilar to the LG and HA groups, which showed a significant improvement.

The results of this study underscore the important role that hyaluronic acid (HA) plays in enhancing the therapeutic efficacy of the lidocaine-loaded thermoresponsive gel (LG) formulation. The high concentration of HA within the LG formulation was found to improve wound closure rates compared with the control treatments significantly. This suggests that HA not only supports the physical process of wound healing but also may influence critical cellular behaviors, such as migration, proliferation, and differentiation, which are essential for effective tissue regeneration.

These data suggest that the LG formulation provides a superior wound healing effect compared with the other formulations, likely because of the synergistic effects of thermoresponsive poloxamer 407 gel, the polyelectrolyte complex (chitosan and pectin), and hyaluronic acid [28]. While lidocaine reduces pain, hyaluronic acid facilitates tissue regeneration and promotes cellular migration [46]. The enhanced wound healing observed in the LG group underscores its potential as an effective treatment for improving palatal wound healing.

#### 2.3.2. Redness

All groups exhibited noticeable redness immediately following wound induction, with an initial severity level of 3 (***), indicating a high degree of erythema caused by tissue trauma [48]. This early inflammatory response is typical following wound creation and reflects increased blood flow to the injury site as part of the body’s natural healing process [49,50]. Over time, the redness progressively subsided in all treatment groups, and by day 5 post-wound induction, erythema had completely resolved. Notably, the groups treated with LG, TA, and HA formulations demonstrated a faster reduction in redness compared with the control and other treatment groups. This suggests that these formulations may exert anti-inflammatory effects, minimizing tissue irritation and promoting a quicker resolution of inflammation. The rapid reduction in redness observed in these groups highlights the therapeutic potential of these gels in modulating the inflammatory response during wound healing.

#### 2.3.3. Edema (Swelling)

No signs of edema were observed on the day the wound was induced, indicating that the initial injury primarily triggered an inflammatory response without significant tissue swelling [51,52]; however, by the first day post-wound induction, moderate swelling was noted in all groups. By day 5, a marked reduction in edema was observed across all treatment groups, and by day 7, no signs of swelling were evident in any groups. Interestingly, after the first day, no further edema was noted across all treatment groups.

This rapid reduction in swelling may be attributable to the anatomical structure of the rat’s palate, which consists of a relatively thin layer of tissue closely adhered to the underlying bone [53,54]. Such unique anatomy might limit visible swelling or make it more difficult to detect edema, even if mild inflammation persists. The tightly bound palatal tissue may also restrict fluid accumulation, contributing to the absence of prolonged edema. This observation emphasizes that the rat palatal wound model may mask certain tissue responses, such as swelling, making it challenging to assess the full extent of inflammatory changes [31,55]. It is essential to consider these anatomical differences when interpreting wound healing and inflammation outcomes in preclinical models. This subtle response highlights the importance of using complementary assessments, such as histological or molecular analysis, to fully capture the inflammatory dynamics and healing processes in these specialized anatomical sites.

#### 2.3.4. Weight, Food, and Water Intake in Tested Rats

Table 3 presents the changes in body weight (g), daily food intake (g), and daily water consumption of rats monitored from Day 0 (prewound induction) to Day 14, providing insights into overall health and recovery during this study.

The initial average weight of the rats on Day 0 was consistent across all groups, ranging from 252 to 258 g. On Day 1, there was a slight decrease in weight across the groups, likely due to the stress of the surgical procedure; however, by Day 5, the weight of the rats showed an upward trend, with notable increases in all groups. By day 14, all groups had achieved significant weight gains, with the LG group reaching the highest average weight of 351 g, indicating healthy recovery and adaptation of the treatment. This suggests that neither the wound induction nor the application of treatment gels significantly impacted the weight of the rats.

Food consumption on Day 0 was similar across all groups, averaging around 15 g. Post-induction, food intake initially dropped on Day 1 but gradually increased as the rats adapted to their treatments. By Day 14, all groups showed increased food intake, with the LG group averaging 15 g, indicating that the treatments did not adversely affect appetite.

Initial water consumption on Day 0 averaged around 60 mL. Similar to food intake, water consumption decreased on Day 1 but gradually increased throughout the treatment period. By Day 14, the water intake of the LG group was notably higher at 64 mL, indicating adequate hydration levels throughout this study.

Overall, these data demonstrate that the test treatments did not significantly impact the rats’ weight, food, and water consumption, reflecting a healthy and stable environment conducive to the evaluation of wound healing.

### 2.4. Histological Analysis

**Figure 4:**
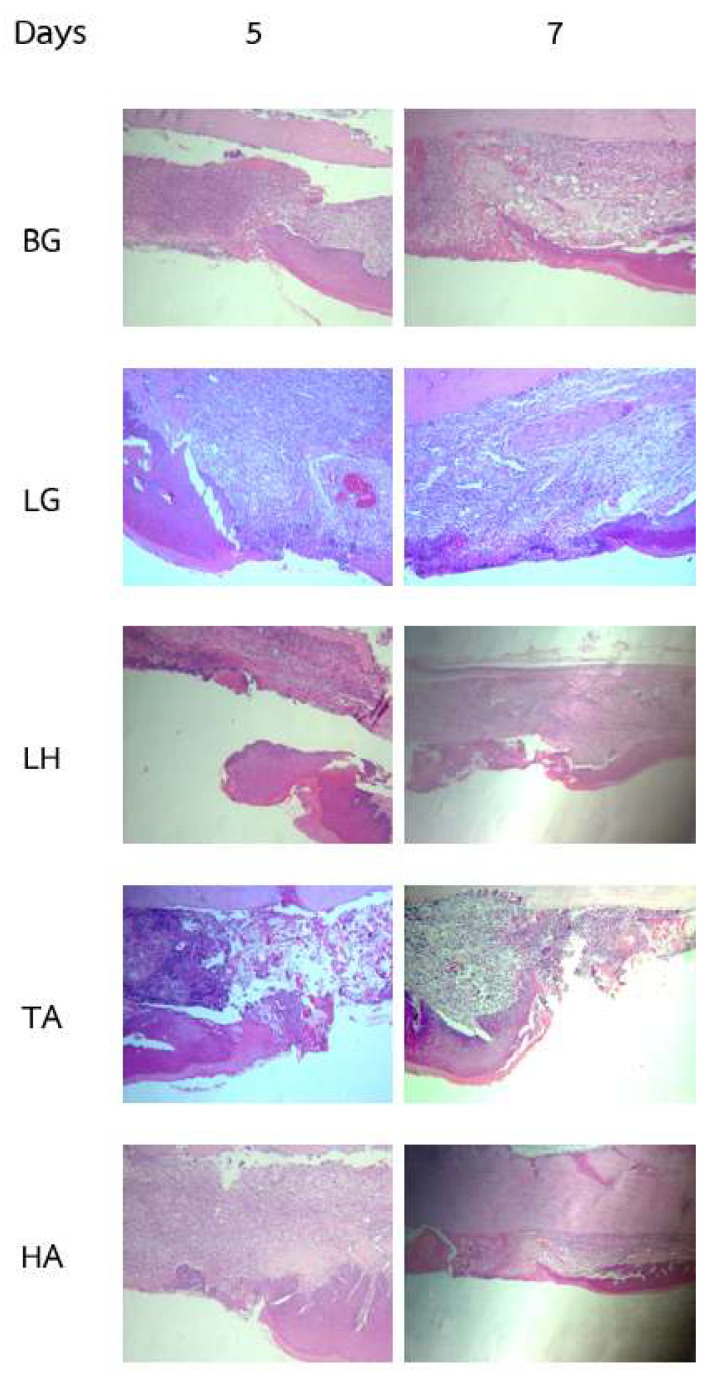
Histological images of the rat palatal wounds on Days 5 and 7 illustrate the reduction in wound size following the daily application of various gels. The treatment groups included a blank control gel (BG), lidocaine-loaded thermoresponsive gel (LG), lidocaine gel (LH), triamcinolone gel (TA), and hyaluronic acid gel (HA). Wound sizes were measured immediately after wound induction and subsequently on Days 5 and 7 to assess the healing progression across different treatments.

The histological analysis of palatal wound healing provides critical insights into the biological processes and tissue responses involved in recovery from injuries such as dry socket. Figure 4 presents histological sections from various treatment groups (BG, LG, LH, TA, and HA) at two individual time points (Days 5 and 7) post-wound induction, illustrating essential aspects of wound healing, including tissue regeneration, inflammatory responses, and overall healing dynamics.

Notable differences in tissue responses across the treatment groups are evident in Figure 4. The LG group demonstrates effective healing, characterized by reduced inflammatory cell infiltration and a more organized tissue structure by Day 7. In contrast, the BG group shows signs of prolonged inflammation or delayed healing, underscoring the therapeutic advantages of active treatments. The TA treatment reveals characteristics indicative of pseudomembranous epithelium, suggesting that it may facilitate healing by forming a protective layer over the wound. This fibrinous exudate serves as a temporary barrier, minimizing infection risk and creating a conducive environment for epithelial regeneration.

The HA treatment also enhances wound healing, as indicated by histological findings. The hydrating properties of HA and its role in the inflammatory response likely contribute to improved cellular migration and proliferation, leading to faster epithelialization and better overall wound-healing outcomes [56]. The presence of HA in the LG formulation may further enhance healing through the combined effects of HA and the thermoresponsive hydrogel. Conversely, the LH group exhibits histological results similar to those of the BG group, indicating a lack of significant improvement in wound healing. This suggests that while lidocaine effectively manages pain as a local anesthetic, it does not possess the regenerative properties observed in TA or HA. Histological analysis of the LH group may reveal persistent inflammation or delayed epithelial regeneration, indicating that lidocaine’s primary role is pain relief rather than promoting the healing process.

These findings highlight the distinct roles of each treatment in palatal wound healing. The lidocaine-loaded gel (LG), TA, and HA formulations demonstrate positive effects on wound healing dynamics by contributing to tissue regeneration and providing protective barriers. In contrast, the efficacy of lidocaine appears limited to its analgesic properties, lacking additional benefits for wound healing compared with the blank control gel. This underscores the necessity of selecting therapeutic agents that not only alleviate pain but also actively promote healing in clinical applications.

While lidocaine is primarily recognized for alleviating pain by blocking sodium channels, recent studies have suggested additional beneficial effects on wound healing. Specifically, lidocaine reduces the release of pro-inflammatory cytokines such as interleukin-6 (IL-6) and tumor necrosis factor-alpha (TNF-α) by inhibiting cytokine release from leukocytes, thereby modulating the inflammatory response and creating a more conducive environment for tissue regeneration [57]. It can also cause vasodilation, enhancing blood flow to the affected area and improving microcirculation in ischemic tissues [58], which ensures a better supply of oxygen and nutrients essential for tissue repair. By stabilizing cell membranes and reducing calcium ion influx, lidocaine minimizes cellular damage in the peri-wound area, preserving viable tissue and promoting faster healing [59]. Furthermore, lidocaine stimulates the proliferation and migration of epithelial cells, such as keratinocytes and fibroblasts, which are critical for re-epithelialization and collagen synthesis during wound healing [60,61]. In our wound scratch assays, cells treated with the LG extract demonstrated increased migration compared with controls, suggesting that lidocaine facilitates epithelial cell proliferation and migration, thereby promoting wound healing; however, in our study, lidocaine did not evidently enhance wound healing compared with the blank gel or the LG, HA, and TA groups.

Histological analysis allows for the identification of various cellular and structural components within the wound area, enabling researchers to assess the impact of different treatments on the healing process. For instance, a well-defined boundary between newly formed tissue and surrounding areas indicates successful epithelialization, while the presence of inflammatory markers, such as neutrophils and macrophages, provides insights into the ongoing inflammatory response and its resolution. Understanding these histological dynamics is crucial for elucidating the pathology of dry socket and identifying effective treatment strategies. This model offers a robust platform for evaluating the efficacy of various formulations while emphasizing the importance of anatomical considerations when assessing healing outcomes. Other models may present challenges in evaluating healing processes due to anatomical differences, which could affect the visibility and accessibility of healing tissue. The histological analysis of palatal wounds allows for a comprehensive understanding of the healing processes, tissue responses, and inflammatory dynamics, which are vital for advancing therapeutic approaches to manage conditions such as dry socket effectively [1,4].

The use of rats in experimental research is widely accepted because of established handling protocols and the ability to minimize suffering. This ethical advantage applies to both palatal and other models; however, the simplicity of the palatal model may reduce the complexity of ethical concerns associated with more invasive procedures.

This research aims to address the current gap in oral wound management by exploring this innovative approach [10,28]. The palatal wound healing model in rats serves as an effective and reliable platform for studying dry socket, particularly because of its ease of use, clear histological evaluation, and reproducibility. While other animal models exist, each with its unique advantages, the palatal model stands out for its direct applicability to the conditions of dry socket and its ability to facilitate targeted research into healing mechanisms and treatment efficacy.

In our study, we developed a thermoresponsive hydrogel incorporating lidocaine, hyaluronic acid (HA), chitosan (CS), pectin (PC), and poloxamer 407 (P407). The findings indicate that the LG significantly accelerates wound healing, evidenced by a statistically significant reduction in wound size compared with control groups (*p* < 0.05). Tissue samples from the treatment group showed increased granulation tissue formation and collagen deposition, highlighting enhanced healing processes.

The hydrogel matrix enhances the wound-healing effects of lidocaine through the synergistic properties of its components. HA plays a crucial role in promoting cell migration and proliferation, angiogenesis, and regulating inflammation [46]. CS also supports hemostasis and tissue regeneration [62,63]. Meanwhile, PC maintains a moist wound environment, which is essential for optimal healing [64]. P407 imparts thermoresponsive properties, allowing the gel to be easily applied as a liquid and then solidify at body temperature, ensuring sustained release of lidocaine and prolonged contact with the wound site [28,65,66].

Although lidocaine itself does not have antibacterial properties, its capacity to promote epithelial cell proliferation and migration significantly enhances wound healing. The combined effects of lidocaine’s anesthetic action and hyaluronic acid’s wound healing result in a cytocompatible formulation that effectively reduces pain while facilitating wound healing by supporting epithelial cell functions.

## 3. Conclusions

Thermoresponsive gels, particularly those formulated with lidocaine and other wound-healing agents, represent a significant advancement in the management of dry socket by effectively addressing both pain reduction and wound healing. The use of a palatal wound model in rats has provided a reliable framework for evaluating the efficacy of these innovative treatments, offering valuable insights into the mechanisms of pain relief and the wound healing process. The unique properties of thermoresponsive gels, combined with the controlled environment of the rat model, enable the development of targeted therapies that can alleviate pain and accelerate recovery, thereby enhancing patient outcomes; however, this study has several limitations that warrant consideration. First, while the rat palatal wound model closely mimics certain aspects of human oral wounds, there are inherent physiological and anatomical differences between rats and humans that may affect the translatability of the results. Second, this study’s duration was limited to the acute phase of wound healing; thus, the long-term effects of the lidocaine-loaded thermoresponsive gel on tissue regeneration and potential chronic side effects remain unexplored. Additionally, the sample size was relatively small, which may limit the statistical power of the findings. For future research, it is essential to investigate the long-term effects of these formulations on tissue regeneration and assess any potential adverse reactions with prolonged use. Expanding studies to include larger sample sizes and diverse animal models will help validate the efficacy and safety of the gels. Furthermore, exploring the application of thermoresponsive gels in other oral wound healing scenarios, such as periodontal surgery or mucosal injuries, will help establish their broader clinical relevance in dental medicine. Ultimately, clinical trials in humans will be crucial to confirm the therapeutic potential of lidocaine-loaded thermoresponsive gels and to facilitate their integration into standard dental care practices.

## 4. Materials and Methods

### 4.1. Preparation of Tested Samples

#### 4.1.1. Preparation of Lidocaine-Loaded Ternary Polyelectrolyte Complex Thermoresponsive Hydrogel (LG)

LG was prepared as previously described [28], with specific attention to ensuring clarity and reproducibility. Initially, stock solutions for each component were prepared. Chitosan (CS) at a concentration of 0.3% *w*/*w* (pKa = 6.5, degree of deacetylation = 55–70%, molecular weight 50,000–190,000 Da; Sigma-Aldrich^®^, St. Louis, MO, USA) was dispersed in 1% *v/v* acetic acid at room temperature (25 °C). The mixture was stirred overnight while maintaining a pH of approximately 4.5 to ensure complete dissolution of chitosan. Separately, pectin (PC) at 0.1% *w*/*w* (high methoxy pectin from citrus peels, pKa = 3.5; Sigma-Aldrich^®^, St. Louis, MO, USA) was dissolved in deionized water at room temperature. Lidocaine hydrochloride (LH; Sigma-Aldrich^®^, St. Louis, MO, USA) was then added to the PC solution to achieve a concentration of 5% *w*/*w*, and the solution was stirred until fully dissolved. The LH-PC solution was added dropwise to the CS solution while stirring at 400 rpm for 5 min at 25 °C to form the initial polyelectrolyte complex (PEC). Subsequently, zinc sulfate (Zn; Fuka, Tokyo, Japan) at a concentration of 0.04% *w*/*w* was incorporated into the mixture. Sodium hyaluronate (HA) at 1.5% *w*/*w* (molecular weight 2.05 × 10⁶ Da; SpecKare™, Nanjing, China) was then added to form the ternary PEC. The PEC mixture was homogenized at 10,000 rpm for 10 min at 25 °C to ensure uniform distribution of all ingredients. In parallel, a 20% *w*/*w* poloxamer 407 (P407; Sigma-Aldrich^®^, St. Louis, MO, USA) solution was prepared using a cold technique and stored at 4 °C for 24–36 h to allow complete solubilization. Finally, the prepared PEC was mixed into the chilled P407 solution, gently stirring to prevent air entrapment and to maintain homogeneity. This process resulted in the final LG formulation containing 5% *w*/*w* LH, with the pH adjusted to range between 6.8 and 7.0.

#### 4.1.2. Preparation of Other Tested Samples Blank Gel (BG)

The blank thermosensitivity gels (BG) were prepared using the same method as LG but without adding LH and were labeled as BG.

The lidocaine gels (LH) were made by incorporating 5% *w*/*w* LH into a P407 solution, followed by stirring with a magnetic bar until a homogenous mixture was achieved. This was labeled as LH for animal studies.

Triamcinolone acetonide gels (TA) were prepared by mixing 0.1% *w*/*w* TA into a P407 solution, stirring until homogenous, and labeled as TA for animal studies.

The HA gels were arranged by loading 1.5% *w*/*w* HA into a P407 solution, stirring until homogenous, and labeled as HA for animal studies.

### 4.2. In Vitro Wound Healing and Cytotoxicity

The biocompatibility of the lidocaine-loaded thermoresponsive gels was evaluated using in vitro cytotoxicity assays. Cell cultures were exposed to varying concentrations of the gels, and cell viability was assessed using standard assays to determine any cytotoxic effects. Additionally, an in vitro wound healing assay was conducted to assess the gels’ impact on cell migration and wound closure. A wound scratch was made on a confluent monolayer of cells, and the rate of wound closure was monitored in the presence of the gels to evaluate its effectiveness in promoting cell migration.

#### 4.2.1. Cell Culture and Preparation

Human gingival fibroblasts (HGF, ATCC no. 5001118, Manassas, VA, USA) were cultured in high glucose Dulbecco’s Modified Eagle’s medium (DMEM, Corning® Life Sciences, Union City, CA, USA) supplemented with 2 mM L-glutamine, 100 U/mL penicillin, 0.1 mg/mL streptomycin, 0.025 mg/mL amphotericin B (Corning® Life Sciences, Union City, CA, USA) and 10% fetal bovine serum (FBS, Corning® Life Sciences, Union City, CA, USA). The cells were incubated at 37 °C with 5% CO_2_ and 95% RH (Shel Lab, Cornelius, Oregon, USA). All experiments were carried out once the cells formed a confluent monolayer, with approximately 2 × 10^4^ cells/well in a 96-well plate or 2 × 10^6^ cells/well in a 10 mm in diameter sterile Petri dish.

#### 4.2.2. Preparation of Extract Solution

Considering that the LG gel transitions from a solution to a gel state at body temperature (37 °C), directly adding the gel to cell cultures would be impractical and could interfere with in vitro assays. To address this issue, we prepared an extract solution derived from the LG gel for use in our experiments.

A measured amount of the LG gel was immersed in Dulbecco’s Modified Eagle Medium (DMEM) without serum. The mixture was incubated at 37 °C for 24 h under gentle agitation to facilitate the release of lidocaine and soluble gel components into the culture medium. During this incubation period, the active ingredients diffused out of the gel matrix and into the medium. After 24 h, the supernatant—now containing the extracted compounds—was carefully collected. To ensure the absence of any residual gel particles that could affect the assays, the extract was filtered through a 0.22 μm syringe filter. This filtration step was crucial to obtain a clear solution suitable for cell culture applications.

The resulting LG extract solution was then used for the subsequent in vitro testing, including the 3-(4,5-Dimethylthiazol-2-yl)-2,5-Diphenyltetrazolium Bromide (MTT) cytotoxicity assay and the wound scratch assay. Using the extract allowed us to assess the biological effects of the released compounds without the physical interference of the gel matrix in the cell culture environment.

#### 4.2.3. In Vitro Cytotoxicity

Predetermined concentrations of the tested samples were incubated with cultured cells for 24 h. Following incubation, each well was treated with 0.5 mg/mL of 3-(4,5-dimethylthiazol-2-yl)-2,5-diphenyl-tetrazolium (MTT, Biobasic, Markham, Ontario, Canada) for 1 h. The cells were then collected and dissolved in 0.05 mL of dimethyl sulfoxide (DMSO, Sigma Aldrich, Shanghai, China). Absorbance at 550 nm was measured using a microplate reader (Varioscan, Thermo Fisher, Waltham, MA, USA). The negative control consisted of untreated cells in the culture medium, while hydrogen peroxide-treated cells (0.1 mg/mL H_2_O_2_) served as the positive control. Cell viability was calculated using the following equation (Equation (1)):

(1) $$Cell\;viability\;(\%)\;=\frac{average\;absorbance\;of\;tested\;sample}{average\;absorbance\;of\;negative\;control\;in\;the\;same\;plate}\times 100$$

#### 4.2.4. Scratch Assay

The scratch assay was conducted to simulate wound healing in a controlled environment. Human gingival fibroblasts (HGF) were cultured to confluence in a sterile 6-well plate (Corning, Bangkok, Thailand). A sterile plastic cell scraper (Gibthai, Bangkok, Thailand) was used to create a perpendicular scratch on the confluent monolayer, ensuring that the cell-free lines intersected at the center. Following the scratches, the test formulations were immediately applied, and the progression of wound closure was monitored over time. For comparison, negative controls received only DMEM, while the positive control group was treated with DMEM supplemented with 10% FBS to provide high-nutrient conditions for optimal healing [67,68]. Cell migration and wound closure were observed at 0, 6, 12, 18, and 24 h, with images captured at each time point. ImageJ software (version 1.54g) was utilized to quantify the wound gap area, providing data on the ability of the formulations to enhance cell migration and promote healing in vitro.

### 4.3. Clinical Wound Healing in Rat Palatal Model

The in vivo efficacy of the lidocaine-loaded thermoresponsive gels (LG) was evaluated using a rat model of palatal wound healing. Full-thickness wounds were created in the palatal mucosa of the rats, after which the LG was applied to the wound site. The rate of wound closure, as well as signs of inflammation and infection, were monitored over time. Key clinical parameters, including wound size, healing time, and tissue regeneration, were recorded to assess the therapeutic potential of LG formulation.

This study received approval from the Institutional Animal Ethics Committee, following procedures outlined in RSU-AEC 003-2564, ensuring compliance with ethical standards [69,70]. Male BrlHan: WIST@Jcl (GALAS) rats, aged 8 to 9 weeks and weighing between 250 and 300 g, were sourced from Nomura Siam International Co., Ltd. in Bangkok, Thailand. The animals were housed in a controlled environment equipped with a Heating, Ventilating, and Air Conditioning (HVAC) system at the College of Pharmacy, Rangsit University, Pratum Thani, Thailand. They had ad libitum access to food and filtered water and were acclimatized in standard Plexiglas cages.

Sample size determination and power analysis were conducted using G*Power software (version 3.1.9.7) [71,72], which indicated that a total of 95 rats were required for the experiment. The rats were housed in cages for approximately one week in a controlled environment, maintaining a 12-h light-dark cycle (lights on at 06:00 h), a temperature of 25 ± 3 °C, and humidity levels of 60 ± 5% RH. Following this acclimatization period, the rats were randomly assigned to their respective study groups.

#### 4.3.1. Palatal Wound Induction

The palatal wound was created following established protocols [31,54]. Prior to administering anesthesia, the working area and equipment were sanitized with 70% alcohol; all necessary tools were systematically organized on the table along with prepared rat cages. Anesthetic dosage was calculated based on the rat’s weight, adhering to a recommended range of 30–40 mg/kg. After the injection, anesthesia was expected to take effect within 15–30 min.

To verify anesthesia, a gentle pinch was applied to the rat’s paw; withdrawal indicated consciousness. Further confirmation involved lifting the rat by its tail, as an anesthetized rat would not attempt to right itself. When placed on its back, the rat should remain still without trying to flip over, while normal breathing was observed to confirm adequate anesthetic depth.

Once anesthesia was successfully induced, the oral cavity was cleaned with saline solution 2 to 3 times, followed by drying the mouth. A 3 mm punch biopsy tool (Kai Medical, Tokyo, Japan) was then used to create a circular wound in the center of the palate with a single press, avoiding repeated pressure to prevent bruising that could impair healing. This procedure was completed within 1–2 min, resulting in a circular wound matching the biopsy punch’s shape.

After wound creation, hemostasis was achieved, and the wound size was photographed and measured using a periodontal probe before applying the test sample. Any rat exhibiting swelling, excessive bleeding, or reduced food and water intake was promptly removed from this study and treated with analgesics, tranquilizers, or other medications as recommended by a veterinarian.

#### 4.3.2. Palatal Wound Treatments

The rats were divided into five treatment groups as follows: Group 1 received a blank control gel (BG); Group 2 was treated with a lidocaine-loaded thermoresponsive gel (LG); Group 3 received a lidocaine gel (LH); Group 4 was treated with a triamcinolone gel (TA); and Group 5 received a hyaluronic acid gel (HA). Following the wound induction procedure, 1 milliliter of the test product was prepared in a syringe and applied uniformly to the wound. This application occurred daily at 9:00 AM for 14 days. During this period, wound sizes, redness, and edema were measured on days 5, 7, and 14 and compared with the initial wound size. Signs of allergic reactions or infections were monitored throughout the testing phase.

#### 4.3.3. Rat Termination and Sample Collection

To establish the baseline group at time zero, five rats were sacrificed immediately after the wound induction. Subsequently, six rats from each treatment group were euthanized on Days 5, 7, and 14 using an overdose of sodium thiopental. Following euthanasia, the maxillae were extracted, and each wound was assessed both clinically through photography and histologically. Palatal specimens were photographed, and the wound area was quantified using ImageJ software (version 1.54g).

#### 4.3.4. Tissue Preparation and Staining for Histological Analysis

Histological analysis was conducted on tissue samples collected from the wound site to examine the cellular and structural changes during the healing process. The samples were stained and analyzed under a microscope to assess the extent of re-epithelialization, collagen deposition, and inflammatory response. This analysis provided detailed insights into the impact of the LG formulation on the wound healing process at the microscopic level.

After photography, specimens were placed in 10% formalin for fixation for a minimum of 24 h. The specimens then underwent decalcification in 10% formic acid for two weeks before preparation for histological analysis. Serial sections of each wound were sliced perpendicular to the palatal midline at the wound’s widest diameter and stained with hematoxylin and eosin. These sections were examined under a light microscope at ×40 magnification, with images captured for documentation.

### 4.4. Statistical Analysis

The statistical significance of these data was assessed using a one-way analysis of variance (ANOVA) followed by Tukey’s test. Categorical variables were expressed as percentages based on sample sizes of 6 and 8 (*n* = 6 and 8). Continuous variables were presented as means with standard deviations (SD), and the normality of these data was verified. Statistical significance (*p* < 0.05) was established using either a Student’s *t*-test or ANOVA to compare mean values.

## Figures and Tables

**Table 1:** Composition of blank control gels (BG) and lidocaine-loaded thermoresponsive gels (LG) formulations, detailing the ingredients: Lidocaine Hydrochloride (LD), Hyaluronic Acid (HA), Chitosan (CS), Pectin (PC), and Poloxamer 407 (P407).

	LD (%)	HA (%)	CS (%)	PC (%)	P407 (%)
BG	-	-	-	-	16
LG1	5	0.5	0.3	0.1	16
LG2	5	1.0	0.3	0.1	16
LG3	5	1.5	0.3	0.1	16

**Table 2:** Assessment of wound size, redness, and edema on days 1, 5, 7, and 14 following treatments with blank control gel (BG), lidocaine-loaded thermoresponsive gel (LG), lidocaine gel (LH), triamcinolone gel (TA), and hyaluronic acid gel (HA).

Groups		BG	LG	LH	TA	HA
Wound size (mm^2^)	Day 5	6.48 ± 0.4	4.27 ± 0.6 ^a^	6.42 ± 0.6	5.98 ± 0.5	5.81 ± 0.4
	Day 7	4.73 ± 0.7	0.96 ± 0.3 ^a^	4.83 ± 0.9	3.72 ± 0.3	3.49 ± 0.6
	Day 14	1.40 ± 0.2	N/S	2.16 ± 0.3	0.96 ± 0.4	N/S
Redness	Day 1	***	***	***	***	***
	Day 5	***	**	***	**	**
	Day 7	**	*	**	*	**
	Day 14	*	N/S	*	*	N/S
Edema	Day 1	**	**	**	**	**
	Day 5	*	*	*	*	*
	Day 7	N/S	N/S	N/S	N/S	N/S
	Day 14	N/S	N/S	N/S	N/S	N/S

Remark: The wound size was measured using the ImageJ software (version 1.54g), which calculates the area of the wound accurately. Redness and edema were assessed visually and graded on a scale: *** representing the highest severity, ** for moderate severity, and * for the lowest severity. The notation N/S indicated normal epithelium without any signs of wound, redness, or edema. Data are presented as the mean ± standard deviation (SD) (*n* = 6). The letter “a” denoted a significant difference (*p* < 0.05) when comparing each group within the same time period.

**Table 3:** Measurements of rat weight (g), food consumption (g), and water intake (ml) before wound induction and during the 14-day treatment period post-induction with blank control gel (BG), lidocaine-loaded thermoresponsive gel (LG), lidocaine gel (LH), triamcinolone gel (TA), and hyaluronic acid gel (HA).

Groups		BG	LG	LH	TA	HA
Weight (g)	Day 0	256 ± 4.2	258 ± 5.6	253 ± 4.8	255 ± 5.4	252 ± 6.1
	Day 1	248 ± 6.3	263 ± 7.4	248 ± 8.1	256 ± 4.4	261 ± 6.4
	Day 5	274 ± 5.4	281 ± 5.2	278 ± 6.4	277 ± 6.0	272 ± 6.7
	Day 7	290 ± 7.3	303 ± 6.0	296 ± 6.2	293 ± 7.1	300 ± 6.6
	Day 14	345 ± 5.2	351 ± 4.5	346 ± 3.8	346 ± 4.2	347 ± 5.0
Food consumption (g)	Day 0	15.6 ± 0.2	15.8 ± 0.4	15.3 ± 0.2	15.0 ± 0.5	15.2 ± 0.6
	Day 1	4.08 ± 0.3	6.33 ± 0.6	4.8 ± 0.7	5.67 ± 0.4	6.12 ± 0.3
	Day 5	5.84 ± 0.5	8.13 ± 0.6	5.96 ± 0.4	7.47 ± 0.6	7.52 ± 0.4
	Day 7	9.20 ± 0.7	10.40 ± 0.3	9.90 ± 0.6	9.22 ± 0.3	10.23 ± 0.6
	Day 14	14.00 ± 0.2	15.00 ± 0.2	14.60 ± 0.3	14.60 ± 0.3	14.74 ± 0.5
Water consumption (mL)	Day 0	60.00 ± 2.4	66.50 ± 4.6	64.50 ± 2.4	64.00 ± 4.2	62.70 ± 5.6
	Day 1	42.00 ± 9.6	50.00 ± 9.3	46.33 ± 8.6	43.33 ± 9.4	46.46 ± 8.3
	Day 5	45.80 ± 8.1	53.8 ± 7.5	55.55 ± 10.3	47.33 ± 9.9	49.00 ± 9.8
	Day 7	46.67 ± 10.9	49.25 ± 10.4	47.33 ± 10.5	50.75 ± 11.3	53.00 ± 11.8
	Day 14	64.00 ± 14.2	64.00 ± 14.2	67.50 ± 15.0	66.50 ± 14.8	67.5 ± 14.4

Remark: Day 0 indicates the day prior to wound induction in rats, while Day 1 to Day 14 corresponds to the post-induction period during which the treatments were administered and monitored.

## Data Availability

The datasets presented in this article are not readily available due to technical and time limitations. Requests to access the datasets should be directed to corresponding author.
